# Surface functionalisation of poly-APO-*b*-polyol ester cross-linked copolymers as core–shell nanoparticles for targeted breast cancer therapy

**DOI:** 10.1038/s41598-020-78601-x

**Published:** 2020-12-10

**Authors:** Rida Tajau, Rosiah Rohani, Siti Selina Abdul Hamid, Zainah Adam, Siti Najila Mohd Janib, Mek Zah Salleh

**Affiliations:** 1 Department of Chemical and Process Engineering, Faculty of Engineering and Built Environment, Universiti Kebangsaan Malaysia, UKM, 43600 Bangi, Selangor Malaysia; 2 Division of Radiation Processing Technology, Malaysia Nuclear Agency, Bangi, 43000 Kajang, Selangor Malaysia; 3 Division of Medical Technology, Malaysia Nuclear Agency, Bangi, 43000 Kajang, Selangor Malaysia

**Keywords:** Cancer, Chemistry, Materials science, Nanoscience and technology

## Abstract

Polymeric nanoparticles (NPs) are commonly used as nanocarriers for drug delivery, whereby their sizes can be altered for a more efficient delivery of therapeutic active agents with better efficacy. In this work, cross-linked copolymers acted as core–shell NPs from acrylated palm olein (APO) with polyol ester were synthesized via gamma radiation-induced reversible addition-fragmentation chain transfer (RAFT) polymerisation. The particle diameter of the copolymerised poly(APO-*b*-polyol ester) core–shell NPs was found to be less than 300 nm, have a low molecular weight (MW) of around 24 kDa, and showed a controlled MW distribution of a narrow polydispersity index (PDI) of 1.01. These properties were particularly crucial for further use in designing targeted NPs, with inclusion of peptide for the targeted delivery of paclitaxel. Moreover, the characterisation of the synthesised NPs using Fourier Transform-Infrared (FTIR) and Neutron Magnetic Resonance (NMR) analyses confirmed the possession of biodegradable hydrolysed ester in its chemical structures. Therefore, it can be concluded that the synthesised NPs produced may potentially contribute to better development of a nano-structured drug delivery system for breast cancer therapy.

## Introduction

Polymeric NPs are not only known as solid colloidal particles with a diameter range of between 1 and 1000 nm; they are also therapeutically utilised as drug carriers due to their macromolecular material contents. They enable the traverse through the blood–brain barrier and prevent a prompt removal of drugs in the lymphatic system^1^. Polymeric nanoparticle products are commonly manufactured in the form of nanospheres and nanocapsules. Furthermore, there are a number of natural-based polymers including polysaccharides, vegetable oils, proteins, and polyesters. They have been extensively developed as the biomaterials for applications such as drug delivery systems, resorbable surgical sutures, and implantable devices for treating human cancer cells. Accordingly, they are non-toxic, inexpensive, easy to design and formulate, and have good stability, biocompatibility, biodegradability, and bioavailability in a targeted delivery compared to synthetic polymer-based NPs^2^. Palm oil is an example of natural polymer that has attracted considerable attention for the development of polymeric NPs due to its biodegradable and biocompatible properties compared to synthetic-based polymers.

Top-down method and bottom-up method are two common methods used for nanomaterial synthesis. In the top-down method, the dispersion of preformed polymers produces the polymer nanoparticles, while in the bottom-up method, the polymerisation of monomers leads to the formation of polymer nanoparticles. This top-down method refers to the techniques that involve physical processing, including cutting, etching, grinding, ball milling, and lithographic. These techniques can be considered as costly, time-consuming, and often result in a broader particle size distribution and do not have proper/precise control of the structure^3^. Meanwhile the bottom-up technique involves both physical and chemical processing methods that include evaporation, sputtering, plasma arcing, laser ablation, chemical vapour deposition, self-assembled monolayers, nanoprecipitation, microemulsion, pyrolysis, and controlled/ living radical polymerisation (C/LRP) such as atom transfer radical polymerization (ATRP), reversible addition-fragmentation chain transfer polymerisation (RAFT), and nitroxide-mediated polymerization (NMP)^4,5^. The bottom-up method is a more common used in synthesising and formulating nanomaterials than the top-down method. This is because the bottom-up method allows the process of polymer synthesis to be controlled based on the chemical properties of the raw material. Moreover, the aforementioned techniques associated with bottom-up method can be possibly used to manufacture novel materials with a narrow distribution of sizes, with much cheaper cost than the top-down techniques^3,4^.

The C/LRP technique has so far been favoured as a potential method used in the aqueous-based dispersed systems due to the proper control of polymer characteristics in terms of its molar mass, molar mass distribution, and macromolecule architecture^6,7^. Although the C/LRP branches into three different techniques, the versatility of the RAFT technology has been found to be a good and modern method for utilisation. This is not only due to its polymerisation conditions allowing the synthesis of polymers with a narrow PDI and high functionality but the method can also be easily conducted at high and room temperatures both in either inorganic or aqueous media. The RAFT polymerisation technology is reportedly used in the synthesis of well-defined smart surfaces and complex molecular structures, such as blocks, brushes and combs, and star copolymers. Moreover, it further allows the free-radical polymerisation to occur in the presence of thiocarbonylthio compounds, such as those of dithioesters, trithiocarbonates, dithiocarbamates, and xanthates^8,9^.

**Figure 1:**
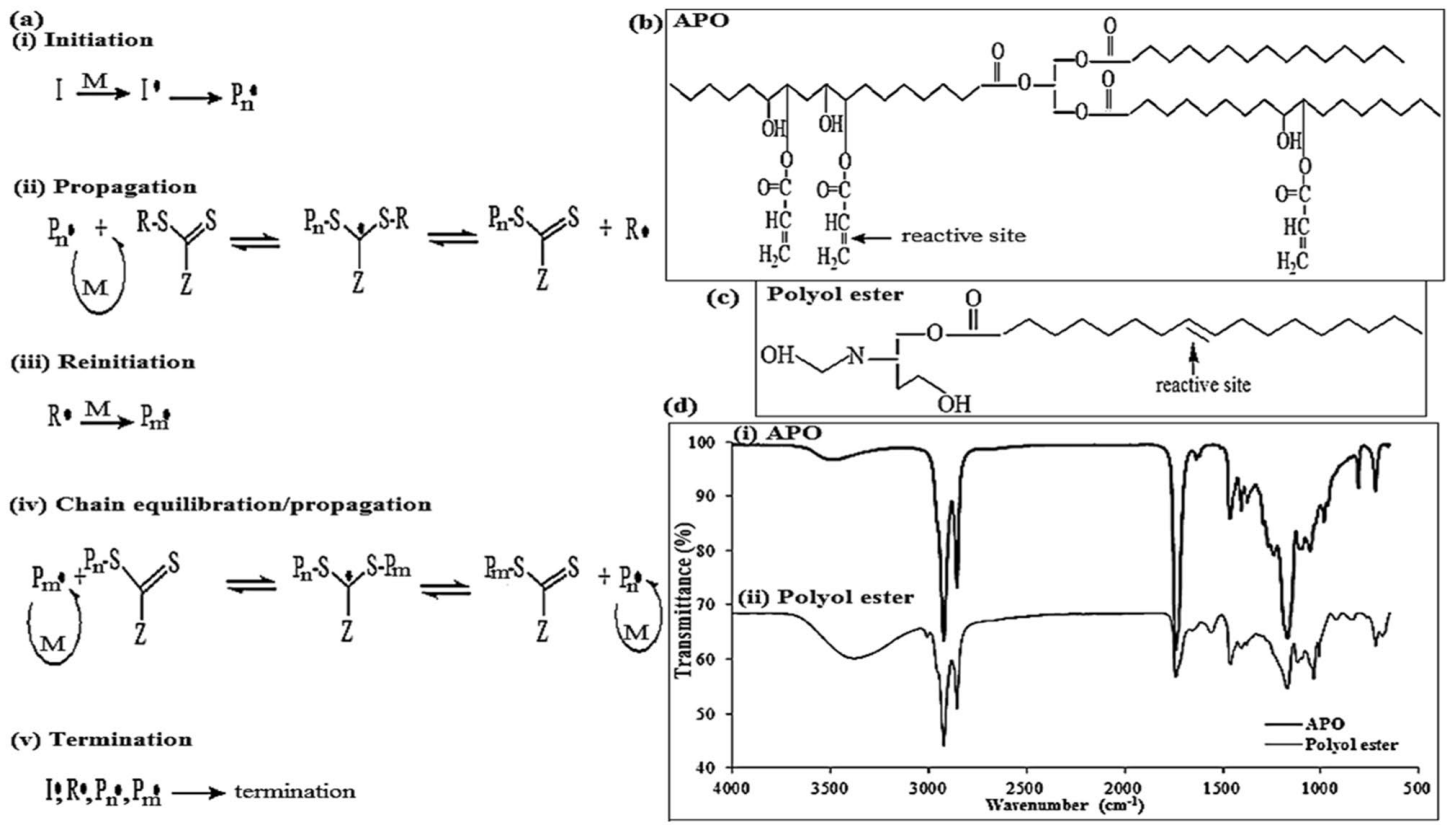
(**a**) The mechanism of reversible addition fragmentation chain transfer (RAFT) polymerisation under thermal, UV, or ionising radiation in the chain extension of macro-CTA agent and in the formation of block copolymer^9^; (**b**) the general chemical structure and products of APO; (**c**) the general chemical structure and products of polyol ester; (**d**) FTIR of the (i) APO and (ii) polyol ester.

The mechanism of RAFT polymerisation under thermal, photoinitiator, or ionising radiation in the formation of macromolecular-chain transfer agent (macro-CTA) and block copolymer is shown in Fig. 1a. As the RAFT mechanism consists of two polymerisation steps, first it begins with the macroinitiator or macro-RAFT agent decomposing into two fragments. This results in the propagation of a polymer chain from the reaction with a single monomer molecule, before continuing with the synthesis of the copolymers. The polymerisation at this stage involves the initiation of the second monomer and the generation of copolymer structures from the incorporated macro-RAFT agent (see Fig. 1a). Accordingly, these well-defined complex macromolecules can also be used to build nanostructures, such as those of micelles, vesicles, and NPs^9^. In particular, gamma radiation as an initiation source is considered to be one of the most powerful tools for generating radicals in RAFT polymerisation. The application of this process offers various advantages, such as easy set-up, inexpensive, less toxic, and an environmentally-friendly process. Therefore, numerous research works have applied RAFT polymerisation for the development of polymeric nanomaterials using gamma radiation^6,7,9–11^.

Furthermore, the most common approach to nanoparticle surface technology is chemical conjugation techniques, with some examples being amine functional group (i.e. including carbodiimide coupling), thiolether functional group (i.e. including maleimide coupling), disulfide, and click chemistry. Specifically, through chemical conjugation, the amine functional group or amide bond seems to be a good choice as it is an easy, simple, and efficient technique to decorate the nanoparticle surface, which can be used for peptide functionalisation. Meanwhile, the advantage of the carbodiimide reaction is the use of non-hazardous reagents and its ability to react in an aqueous solution and at room temperature^12^. The toxicity of the reaction is estimated to be weak as the 1-Ethyl-3-(3-dimethylaminopropyl) carbodiimide (EDC) is converted to a non-harmful urea derivative in a coupling reaction^13^. The targeting ligands are otherwise known as monoclonal antibodies (mAbs), antibody fragments, nucleic acid (aptamers), proteins, peptides, small molecules (i.e. folic acid, galactose, estradiol, and biotin), and others (i.e. vitamins and carbohydrates). These ligands can be paired with NPs to improve the efficiency of delivery^14^. Accordingly, peptides are attractive targeting molecules due to their small size, low immunogenicity, good stability, high specificity, low toxicity, fast manufacturing at low cost and nanoparticle conjugation, and elevated success rates in clinical trials^15^. The cell-targeting peptides (CPTs) interact in cells or tissue directly, i.e. arginylglycylaspartic acid (RGD) have thus received consideration from many research groups for its potential use in breast cancer drug delivery as surface ligands^16^. The strategy towards applying drugs in targeted anticancer therapy is by permitting them to accumulate the drugs in NPs, whether externally and/or internally.

A previous work has stated that the production of micro/nano-particles created from APO and a dispersed surfactant method formed by a typical technique of radical polymerisation, resulting in a broad distribution of particle size, which is an undesirable property^17^. Therefore, this work attempts to synthesise poly(APO-*b*-polyol ester) NPs using APO and polyol ester as shown in Fig. 1b,c via low-dose gamma radiation-induced RAFT polymerisation and cross-linking processes on the synthesis of potential drug carriers^18,19^. The infrared (IR) spectra of the APO and the polyol ester are shown in Fig. 1d. This work also demonstrates the physicochemical properties of NPs obtained by gravimetric analysis, dynamic light scattering (DLS), gel permeation chromatography (GPC), and ultraviolet–visible (UV–Vis) spectrophotometer, as well as characterisations via FTIR and NMR. It is expected that the gamma radiation-induced RAFT technique can be a successful method for the formulation and synthesis of core–shell NPs. A mean diameter of less than 300 nm and a small particle size distribution should be obtained in order to modify the surface of the NPs with peptide in the specific delivery of paclitaxel for breast cancer therapy.

## Results and discussion

### Formation of poly(APO-*b*-polyol ester) nanoparticles

**Figure 2:**
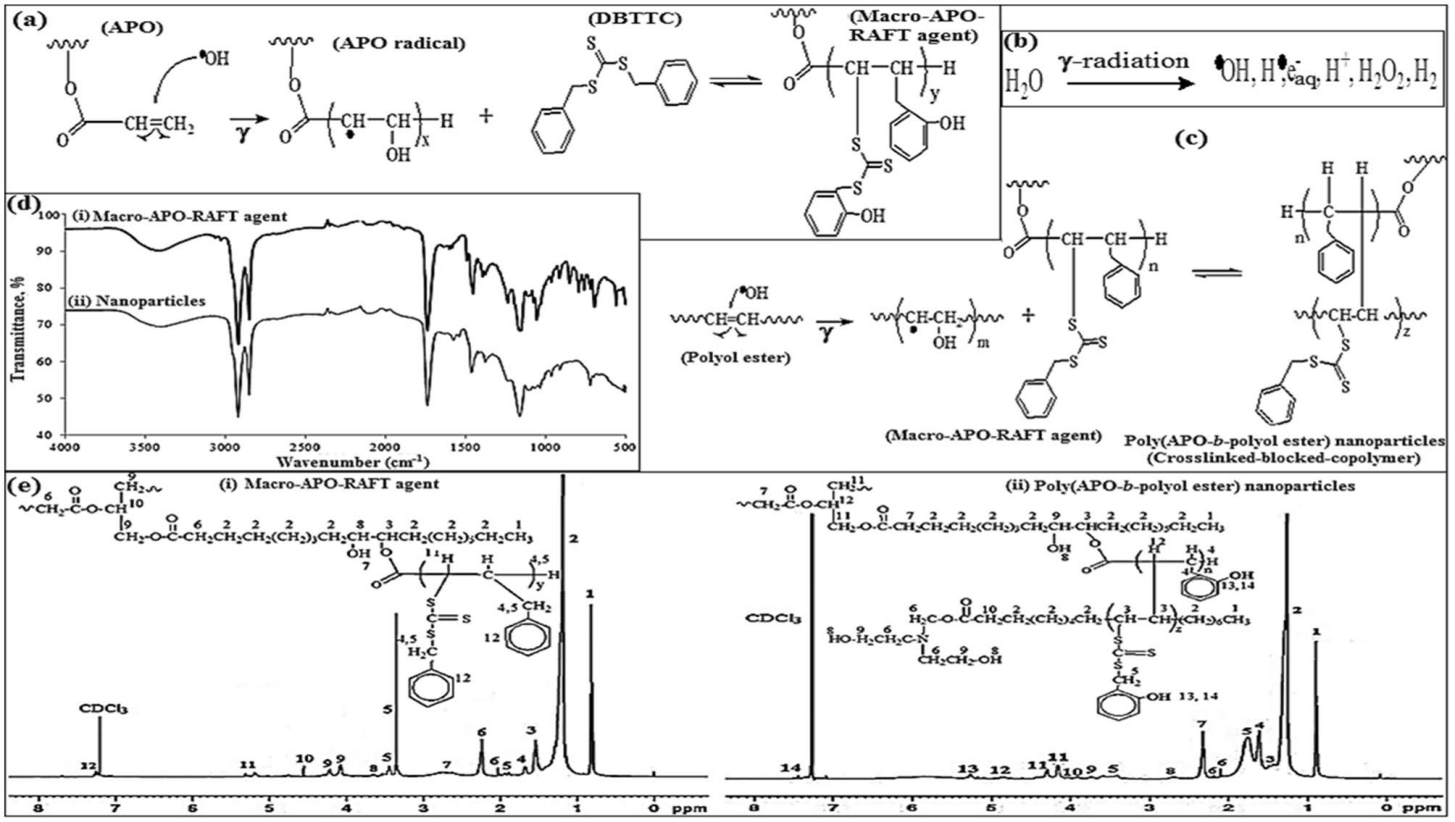
(**a**) Mechanism of reversible addition fragmentation chain transfer (RAFT) polymerization of macro-APO-RAFT formation; (**b**) the mechanism of water radiolysis by the gamma radiation; (**c**) mechanism of reversible addition fragmentation chain transfer (RAFT) polymerization of poly(APO-*b*-polyol ester) nanoparticle formation; (**d**) FTIR of the macro-APO-RAFT agent and the poly(APO-*b*-polyol ester) nanoparticles. (**e**) ^1^H-NMR of (i) the macro-APO-RAFT agent and (ii) the poly(APO-*b*-polyol ester) nanoparticles.

The macro-CTA, namely macro-APO-RAFT agent is synthesised via the RAFT polymerisation method with the use of the APO synthesised from palm olein. Figure 2a shows the schematic reactions of macro-APO-RAFT agent formations. The pre-formed polymer or macro-APO-RAFT agent was first prepared by exposing the microemulsion system to 500 Gy of gamma irradiation, followed by the gamma radiation energy absorption of the water molecules under the pulse radiolysis technique^20,21^.

As a result of exposure to the gamma irradiation, the formation of the initial reactive intermediates such as those of the hydroxyl radicals (∙OH), hydrogen atoms (H∙), hydrated electrons (e^−^), hydrogen ions (H^+^), peroxide (H_2_O_2_), and hydrogen (H_2_) from the water molecule (H_2_O) hydrolysis is used in the preparation of the macro-APO-RAFT agent (Fig. 2b). Among all of these reactive radicals, the hydroxyl radical is considered as responsible for the reactivity transfer from the water to the polymer^22,23^. This is due to many previous studies having attested to its better effectiveness in the radiation-induced polymerisation process compared to those of the hydrogen atoms^24^. Furthermore, the reaction between the hydroxyl radical and the carbon double bond addition in the chemical structure results in the formation of radical sites on the APO chains for synthesising macro-APO-RAFT agent with the presence of DBTTC (Fig. 2a).

Next, the cross-linked-copolymer NPs or poly(APO-*b*-polyol ester) are produced by adding the polyol ester (i.e. second monomer) into the macro-APO-RAFT agent system. Under this circumstance, the initiation reaction is similar to the scheme reaction shown in Fig. 2b, where the water molecules undergo a radiolysis from the reactive radical irradiation. This is followed by the hydroxyl radical reaction with the polyol ester of its carbon double bond, forming the polyol ester radical (Fig. 2c)^25^. The polyol ester-radical then reacted with the macro-APO-RAFT agent in the formation of cross-linked-blocked-copolymers with a thiocarbonyl thio end-group, named by poly(APO-*b*-polyol ester) NPs.

The use of the radiation-induced RAFT polymerisation approach for synthesising the copolymer nanoparticles in the present study can be claimed as safe, conducted in a controllable process at room temperature, faster, and involved no catalyst to perform the reaction compared to the thermal method and/ or other C/LRP methods such as the NMP process that requires the presence of both high-temperature reaction and ATRP-based catalyst^26^. Furthermore, the application of gamma radiation as a tool in RAFT polymerization is a promising initiation source for the formation of copolymer nanoparticles with a defined molecular weight and narrow molecular weight distribution (see Table 1)^8,10,27^. RAFT polymerisation is applied in the manufacturing of multi-block copolymers and complex polymer architectures due to its living and controllability throughout the polymerisation process. Therefore, the RAFT polymerization method can be an alternative and promising technique for the development of polymeric nanoparticles.

**Table 1:** Transformation of particle size of poly(APO-*b*-polyol ester) NPs under different gamma irradiation doses and their number average molecular weight, the polydispersity index, and the gel fraction of the poly(APO-*b*- polyol ester) NPs from the irradiation of the low gamma doses.

Dose, Gy	Hydrodynamic particle diameter, nm	Number-average molecular weight (Mn), kDa	Polydispersity index (PDI)	Gel fraction, %
0	92.47 (± 0.50)	–	–	–
100	95.12 (± 5.09)	22.17	1.0590	57.97
400	89.81 (± 6.63)	20.84	1.0245	88.11
700	142.09 (± 0.97)	23.75	1.0099	95.22
1000	100.16 (± 9.45)	–	–	–
5000	99.03 (± 0.58)	–	–	–
10,000	94.50 (± 2.05)	–	–	–

### Fourier transform infrared (FTIR) spectroscopy

The infrared (IR) spectra of the macro-APO-RAFT agent and the poly(APO-*b*-polyol ester) nanoparticles are shown in Fig. 2d. Referring to the first irradiation process performed in the formation of the macro-APO-RAFT agent, the occurrence of the new IR peaks at 2658, 2139, 2084, 1675, 1632, 1603, 1395, 1399, 1241, 988, 950 and 855 cm^−1^ on the FTIR spectroscopy corresponded with the thio, sulphate, aromatic-alkene, and benzene functional groups (Fig. 2d(i)). They were found to represent the interaction between the APO and respective DBTTC molecules. Due to the absence of these new IR peaks on the original APO spectra (Fig. 1d(i)), these new IR peaks thus confirmed the appearance of a pre-formed polymer with thiocarbonyl thio end group; in this case, this was the chemical structure of a macro-APO-RAFT agent (Fig. 2d(i)).

The use of an FTIR spectroscopy in the second irradiation process for the formation of poly(APO-*b*-polyol ester) nanoparticles further verified the appearance of an amine (C–N) functional group at the wavenumber of 1000 and 1028 cm^−1^. The thio (2689 cm^−1^), isothiocyanate (2140, 2020 and 1250 cm^−1^), aromatic-alkene (1588, 1562 and 940 cm^−1^) and benzene functional groups (1380 cm^−1^) in Fig. 2d(ii) signifies the formation of the poly(APO-*b*-polyol ester) nanoparticles accordingly^19^. Supplementary Table S1 shows the details on the assignment of IR peaks for macro-APO-RAFT agent and poly(APO-*b*-polyol ester) nanoparticles. Assignments of the four major functional groups such as thio, sulfate, aromatic-alkene and benzene were still present after being copolymerised by poly(APO-*b*-polyol ester) nanoparticles. This signifies the effective reaction by the RAFT technique for the formation of NPs between the APO and the polyol ester (see Supplementary Table S1).

### Nuclear magnetic resonance (NMR)

Detailed properties and locations of the proton-NMR shifts (ppm) for the macro-APO-RAFT agent and the poly(APO-*b*-polyol ester) nanoparticles have been shown in Supplementary Table S2. Therefore, the use of a ^1^H-NMR spectroscopy revealed the formation of a macro-APO-RAFT agent from the thio-sulfonate sulphide and benzene proton signals that occurred at the respective 1.672 ppm, 1.895–1.944 and 3.426–3.529 ppm for the thio-sulfonate sulphide group and 7.270–7.265 ppm for the benzene group. The spectra of the macro-APO-RAFT agent exhibited in Fig. 2e(i) further verified the formation of a molecular structure between the macro-APO-RAFT agent and the poly(APO-*b*-polyol ester) nanoparticles in Fig. 2e^28^.

Meanwhile, the formation of the poly(APO-*b*-polyol ester) nanoparticles was found to be hindered by the absence of the C–N peak in the macro-APO-RAFT spectra as shown in Fig. 2e (i). Therefore, the amine protons (–CH_2_–N) and the carbon protons next to the ester linkages (COO–CH_2_–) or the presence of a molecular polyol ester occurring at 2.100–2.200 ppm and 3.7000–3.950 ppm in Fig. 2e thus (ii) verified the formation of the poly(APO-*b*-polyol ester) nanoparticles from a copolymerisation between the macro-APO-RAFT agent and the polyol ester. The disappearance of certain sulphide peaks at 3.426–3.529 ppm is also indicative of the sulphide group shift^28^.

Further confirmation of the copolymerisation process showed by the appearance and shifting of the benzene peaks at 7.300–7.500 ppm in the ^1^H-NMR spectra region. This was possibly be due its associated chemical structure in the poly(APO-*b*-polyol ester) nanoparticles system^28^.

### Particle size

The NPs increased in size throughout the radiation process until they received sufficient radiation energy to complete the copolymerisation (see Table 1). The optimum dose for the copolymerised NPs was determined by the transformation in their sizes throughout the irradiation process. The ideal dose for the copolymerised NPs is defined directly from the irradiation dose right before the NPs decrease in size (see Table 1). Their hydrodynamic size was less than 200 nm. The NPs produced possess a common polymeric particle size for drug delivery, as previously reported in many studies (see Table 1)^2,29^.

The zeta potential results revealed that the surface charge of the NPs at an optimum dose of 700 Gy had a highly stable value of 61.97 mV in a colloidal system. The resulting NPs also possessed a positive surface charge as indicated by the zeta potential measurement, thereby indicating the interaction of the CTAB molecules surrounding the NPs with the aqueous molecules. As reported in a few studies, positively-charged or cationic-type polymeric NPs are perfect for cancer therapy applications due to their advantages over the commonly used liposomes in drug delivery systems. For instance, cationic NPs are more stable and offer more protection during cellular trafficking^30^.

**Figure 3:**
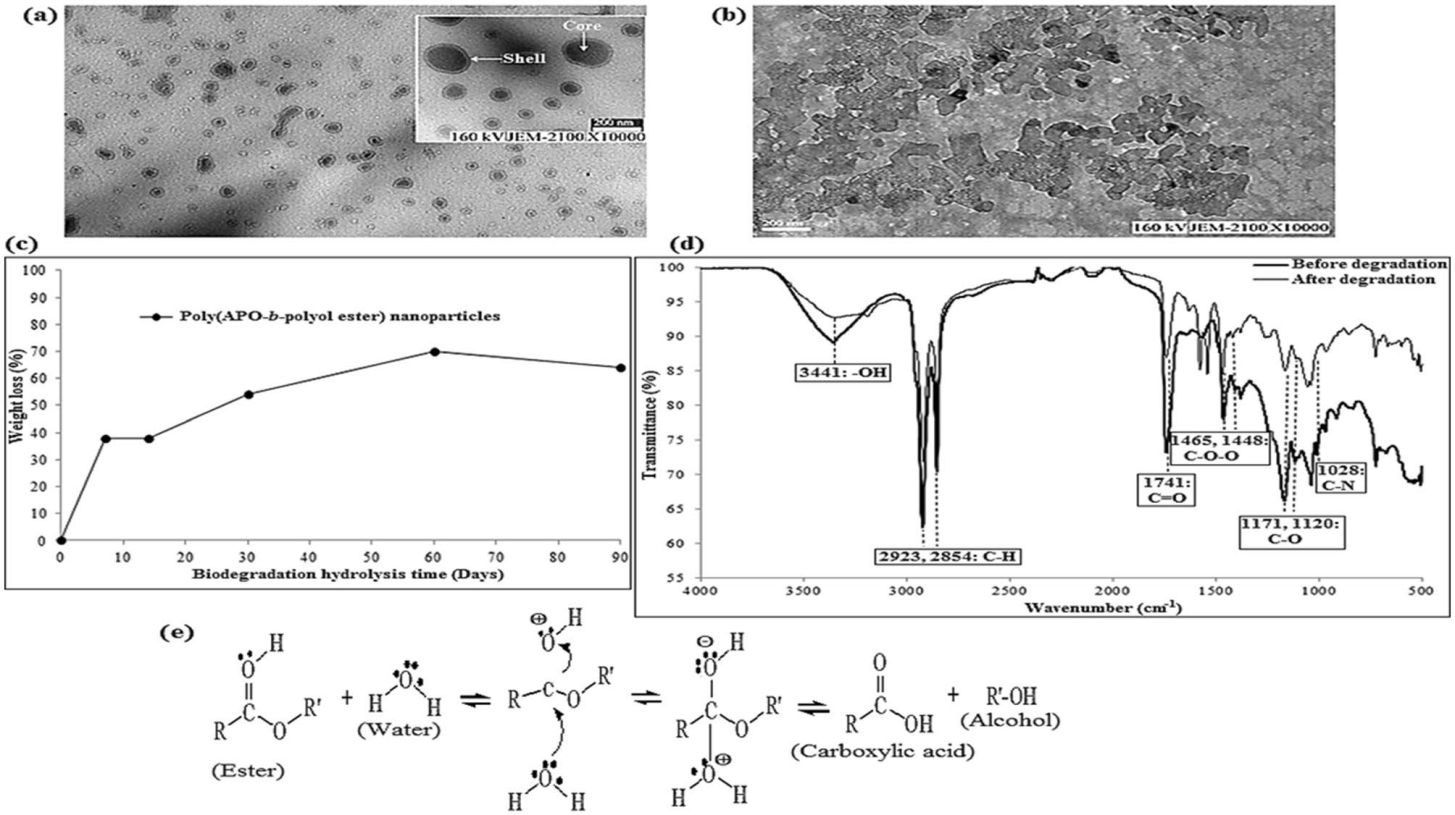
TEM micrograph of (**a**) poly(APO-*b*-polyol ester) (**b**) after degradation for 90 days, in the SBF solution; (**c**) weight loss of poly(APO-*b*-polyol ester) nanoparticles in SBF solution as a function of degradation time in SBF for 90 days at 37 °C; (**d**) FTIR spectra of poly(APO-*b*-polyol ester) nanoparticles before and after 90 days degradation process; (**e**) hydrolysis reaction of ester bonds in water.

Therefore, this study revealed that the gamma radiation-induced RAFT polymerisation could generate the desired polymeric NPs suitable for drug delivery applications. The copolymerisation of APO and polyol ester was successfully conducted in a colloidal system to obtain spherical core–shell nano-scale sized particles (Fig. 3a).

### Effect of the radiation dose on the molecular weight and gel fraction of nanoparticles

In reference to Table 1, the increase in the gel fraction was found to correlate with the copolymerisation reaction of the designed NPs at the hydrodynamic particle diameter of 142.09 nm. Since the results showed the gel fraction percentage of the NPs to be increasing with the radiation dosage, this confirmed the copolymerisation and crosslinking density for inducing their size.

As most of the synthesised NPs are formed by the crosslinking polymerisation, they are produced by the combination of two processes, namely the intramolecular crosslinking and intermolecular crosslinking^22,31^. The intermolecular crosslinking causes an increase in the average size of the polymer chains (molecular weight) and can contribute to the creation of macroscopic or branched structures. In contrast, the intramolecular cross-linking leads to the formation of additional bonds between the single macromolecule segments or a closed-loop structure with a smaller dimension^31^.

Similarly, a larger diameter of the NPs was observed at 700 Gy compared to the 0 Gy and an increase in the number average molecular weight of the obtained NPs at 700 Gy with 23.75 kDa are shown in Table 1. This indicated the copolymerisation and intermolecular crosslinking process to dominate the recombination of molecules in the formation of the poly(APO-*b*-polyol ester) NPs, leading to the increased particle size^9,31^. Besides, RAFT polymerization often allows nanoparticles to be synthesized with well-controlled molecular weight distribution and narrow polydispersity index (PDI) when the necessary radiation dose is acquired to crosslink the block copolymers as nanoparticle structures such as from the irradiation of the low gamma doses (Table 1).

### In vitro degradation

Next, the biodegradation of the poly(APO-*b*-polyol ester) nanoparticles was studied in an SBF solution at a temperature of 37 °C. The results are displayed in Fig. 3c, whereby the NPs show a good reaction towards weight loss in the SBF solution. The weight loss calculated (using Eq. 1) for the NPs was found to be 70% and 64% at the respective analysis periods of 60 days and 90 days. This result indicated that the nanoparticle substances had the capability to gradually decompose in the SBF solution, which was supported by the nanoparticle sample degradation. This can be measured using FTIR spectroscopy under dry conditions (refer to Fig. 3d). The FTIR spectra showed specific changes in several peak intensities of the NPs’ chemical functional groups both before and after the degradation process. It was observed that around seven peak intensity bands of the functional groups decreased after the degradation process. This decrease in the FTIR spectral peaks indicated that the weight loss was mainly due to the degradation of the hydrolysable ester bonds^19^ and some of the other functional groups such as the hydroxyl, alkyl, thio and amine groups in the SBF solution. The details of these degraded peaks were as follows: (1) O–H of hydroxyl group at 3441 cm^−1^, (2) C–H of alkyl group at 2923 and 2854 cm^−1^, (3) S–H of the thio group at 2689 cm^−1^, (4) C=O of the carbonyl at 1741 cm^−1^, and (5) C–N of amine group at 1028 cm^−1^, as well as the (6) C–O–O at 1465 and 1448 cm^−1^, and (7) C–O at 1171 and 1120 cm^−1^ of the ester groups. The FTIR results demonstrated the decay of the chemical structures in the NPs and implied their inherent biodegradation property.

Several studies have found that NPs in the SBF solution are degraded by ester bonds, resulting in monomer separation, decomposition of polymer chains, and dissolution of degradation products. This mechanism leads to the formation of small water-soluble fragments of carboxyl end-group chains^32^ (Fig. 3e). Meanwhile, the TEM micrographs in Fig. 3b show that the poly(APO-*b*-polyol ester) nanoparticles structure collapses slightly after the degradation process compared to its better shape prior. This showed that the fresh NPs were well-dispersed in the SBF solution with no aggregation occurring before storage period (Fig. 3a). The TEM images presented in Fig. 3b further confirm that the degraded NPs deform into complex agglomerated particles after 90 days of degradation. This image indicated that the poly(APO-*b*-polyol ester) NPs with a molecular weight of 23.75 kDa were able to retain their degradation property. Furthermore, polymeric nanocarriers with molecular weights of below 30–50 kDa are often chosen as the backbone for drug delivery system because these polymers are highly likely do not elicit any toxic responses and are suitable for renal clearance^33–35^. Hence, this property could become an added advantage for the NPs as potential biodegradable nanocarriers if they were to be applied in a drug delivery system.

### Surface functionalisation of nanoparticles with peptide

**Figure 4:**
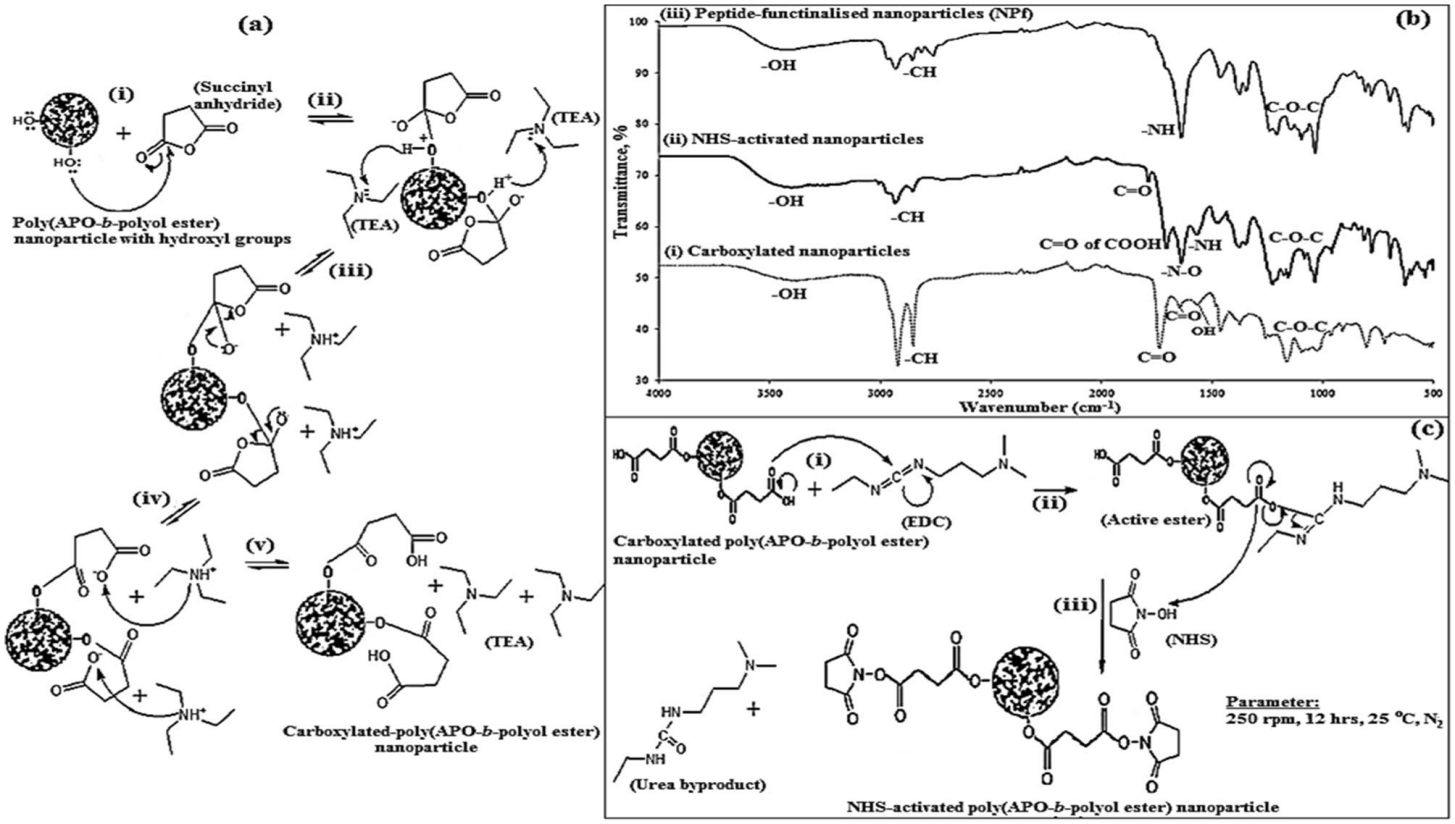
(**a**) Carboxylation of hydroxyl group to carboxyli group. (**b**) FTIR spectra of (**a**) carboxylated nanoparticles (**b**) NHS-activated nanoparticles, and (**c**) peptide-functionalised nanoparticles. (**c**) NHS activation process of carboxylated-nanoparticle.

Succinylation method using succinyl anhydride was used to transform the hydroxyl group of the NPs into a carboxyl group in the presence of DMAP and TEA^36^. After the succinylation process, the yellowish solid colour of the poly(APO-*b*-polyol ester) NPs reformed into a dark yellow solid colour. The schematic reaction of transforming the hydroxyl group of nanoparticles to carboxylated-nanoparticles is shown in Fig. 4a. FTIR spectra analysis in 4b shows variable peaks of hydroxyl (–OH) at 3494 cm^−1^ and 1623 cm^−1^, and carboxylic acid (–C=O) at 1709 cm^−1^. This implicates the existence of carboxylic absorption on the nanoparticle’s molecular structure. In addition, strong ester absorptions i.e. (C=O) at 1739 and 1709, and (C–O–C) at 1246, 1170, 1113, 1097 and 1058 cm^−1^ both confirmed the successful functionalisation of carboxylic molecules onto the NPs (Fig. 4b)^37^. Supplementary Table S1 reveals the transformation of IR functional groups for neat NPs into carboxylated NPs in detail.

Meanwhile, NHS-activation process by using NHS and EDC was utilised to modify the carboxylic group of NPs to the NHS group (see Fig. 4c). Accordingly, the amine coupling with the techniques of EDC/NHS is a strategy for peptide conjugation^15,38–42^. From the FTIR spectral analysis shown in Fig. 4b, several new peaks representing the NHS molecule appear in the NHS-activated nanoparticles. For example, an ester group (C=O) appeared at 1789 cm^−1^, while amide groups i.e. (N–H) and (C=O) were found between 3700–3500 and 1715–1585 and 879 cm^−1^, respectively^37^. Furthermore, Supplementary Table S1 presents a comparison of the IR peak and functional groups between the carboxylated-nanoparticle and NHS-activated nanoparticle. The latter NPs were effectively synthesised with carboxylic group consumption and subsequently substituted by the NHS molecules in the nanoparticle structure. This demonstrates the efficacy of an EDC-mediated reaction by producing an intermediate sulfo-NHS ester product or the NHS-activated nanoparticles.

**Figure 5:**
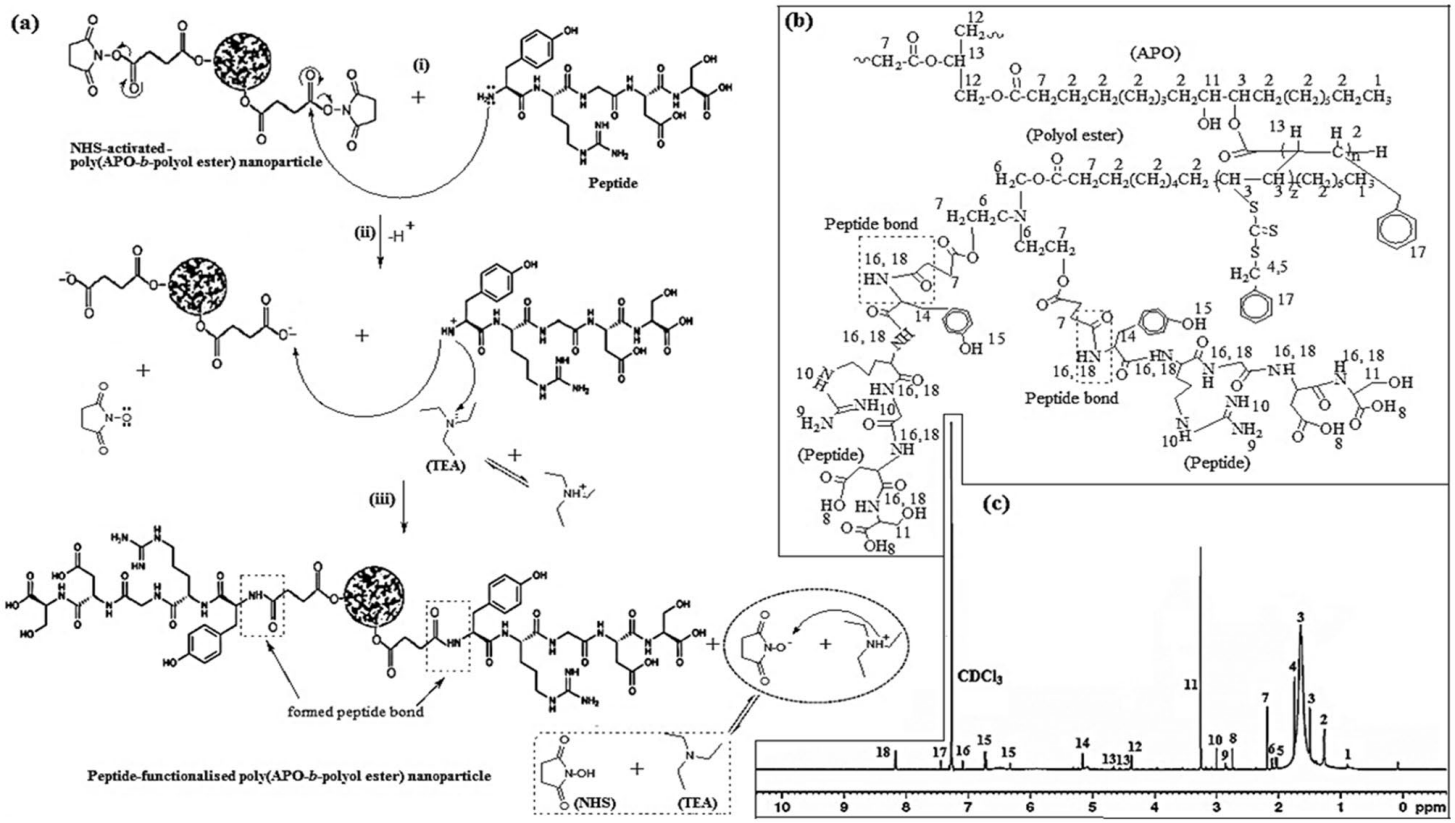
(**a**) Functionalisation process of NHS-activated nanoparticles with peptide; (**b**) peptide-functionalised-poly(APO-*b*-polyol ester) nanoparticles, (NPf nanoparticles); (**c**) ^1^H-NMR spectra of Peptide-functionalised-poly(APO-*b*-polyol ester) nanoparticles (NPf).

The peptide was conjugated with NPs in the presence of DMAP and TEA to improve the efficacy of their therapeutic delivery to target cells^43^. The schematic process for the peptide functionalisation to the NHS-activated nanoparticles is presented in Fig. 5a. The peptide’s amine (–NH_2_) group acts as a nucleophile and targets NPs ester (–C=O–O–) as it is a strong electrophilic agent, thus allowing nucleophiles to easily attack [Fig. 5a(i)]. Meanwhile, the TEA then deprotonated the positively-charged peptide cation (or amine), followed by the NHS removal process [see Fig. 5a(ii)]. The application of DMAP as a catalyst towards the reaction mixture is intended to increase the coupling efficiency^43^. Following this, the peptide bond is developed to form peptide-functionalised poly(APO-*b*-polyol ester) nanoparticle, whereby subsequent TEA protonation for the oxygen anion of NHS produces their side products [see Fig. 5a(iii)].

From the analysis of FTIR spectra in Supplementary Table S1, the NHS ester molecule (C=O) at absorption peak 1789 cm^−1^ disappears upon the functionalisation of the peptide. As a result, the ligand molecule of the peptide was successfully functionalised to the NHS-activated nanoparticles. Broad and strong alcohol peak absorption occurred at wavenumber 3491 cm^−1^ for NPf sample formulation compared to the NHS-activated nanoparticles [Fig. 4b(ii, iii)]. Besides, the alkyl, amide, and phenol groups were found to appear at the respective wavenumbers of 2848, 1645 and 1370 cm^−1^, thereby representing the presence of peptide molecule in the sample NPf^44^.

Disappearance of ester and nitro from the spectra indicated that the peptide was conjugated with the NHS group of NPs (Fig. 4c). Furthermore, the ester peaks (–C–O–C–) between 1230 and 988 cm^−1^ almost disappeared or shifted, which suggested the peptide coupling to the nanoparticle surface^45^ [Fig. 4b(iii)]. Supplementary Table S1 shows the IR peak and functional groups of peptide-functionalised nanoparticles compared to the NHS-activated nanoparticles for usage in evaluating the effective peptide synthesis conjugated to the NPs.

Figure 5b,c display the chemical molecular structure and ^1^H-NMR proton of peptide-functionalised nanoparticles, respectively. The presence of peptide compounds in the NPf nanoparticles is confirmed by the new chemical shift of carboxylic acid (–C=O–OH) at 2.744 ppm, primary amine (–NH_2_) at 2.854 ppm, secondary amine (-NH) at 2.998 ppm, amine-N-Hydroxyl (–NH–CH_2_–Phenol) at 5.136–5.171 ppm, phenol at 6.325 ppm and 6.716–6.728 ppm, and carboxylic amide (–C=O–NH–) at 7.10 ppm and 8.159–8.181 ppm (Fig. 5c, see Supplementary Table S2)^46^. Meanwhile, arginine-glycine-aspartate (Arg-Gly-Asp) or RGD peptides are the ligands for vβ3 and αvβ5 integrins that can act as cell adhesion sites^47^. Hence, the spectra in the ^1^H-NMR showed that at 8.159–8.181 ppm and 7.10 ppm, the NPfTX nanoparticles were actually coupled with RGD peptides carrying amide-Arg^46^. All of these peaks were, however, undetected in neat NPs. These spectral variations in ^1^H-NMR are thus consistent with the development of amide bonds coupled to the peptide on the NPs [Fig. 5a(iii)]. Based on the NMR and FTIR analyses, the molecular structure of NPf nanoparticles is identified and shown in Fig. 5b. As a result, the NPf nanoparticles have a high functionalisation of the peptide at 96.13% and, about 99.60% of NHS molecules have been consumed by peptide conjugation to the NHS-activated nanoparticles. Results showed that significant functionalized efficiency of peptides on the surface treatment of nanoparticles.

### Paclitaxel loaded nanoparticles

**Figure 6:**
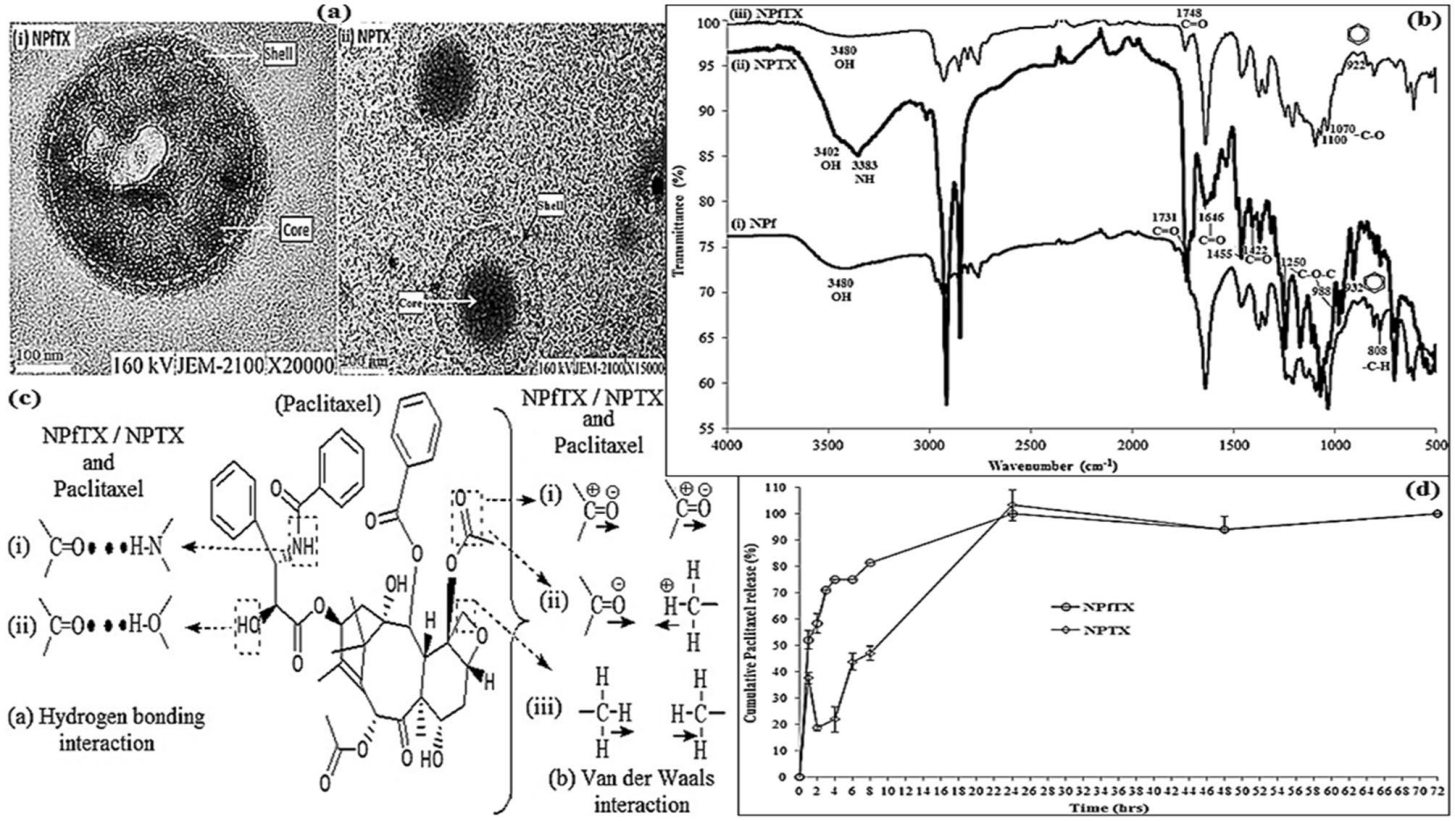
(**a**) TEM image of core shell poly(APO-*b*-polyol ester) nanoparticles: (i) peptide-functionalised nanoparticles with paclitaxel (the NPfTX) and (ii) non-functionalised nanoparticles with paclitaxel (the NPTX); (**b**) FTIR spectra of (i) NPf, (ii) NPTX, and (iii) NPfTX nanoparticles; (**c**) the non-covalent reaction proposed between the NPs and paclitaxel; (**d**) in-vitro release study of NPfTX and NPTX nanoparticles.

It was noted that when loaded with paclitaxel, the mean NPf nanoparticle size increased continuously from 239.33 nm (± 9.21) to 263.08 nm for the development of NPfTX nanoparticles as expected^48^. For non-functionalised nanoparticles, NPTX increased to 176.99 nm after paclitaxel loading (Table 2). The TEM images result in a spherical nanoparticle core–shell for NPfTX and NPTX as shown in Fig. 6a.

**Table 2:** Particle size and zeta potential of the NPs and their nanoparticles yield, the drug loading content, the entrapment efficiency, the in vitro drug release kinetic values, and the diffusion exponent values of the peptide-functionalised and non-peptide functionalised poly(APO-*b*-polyol ester) nanoparticles, loaded with paclitaxel.

Sample	NPfTX	NPTX
Particle diameter (nm)	263.08 (± 1.59)	176.99 (± 14.44)
Zeta potential (mV)	12.12 (± 1.60)	16.33 (± 2.14)
Yield (%)	52.87	62.92
Drug Loading (%)	7.12	2.9
Entrapment efficiency (%)	100	100
Zero order (R^2^)	0.8074	0.9741
First order (R^2^)	0.9871	0.9729
Higuchi (R^2^)	0.9198	0.9798
Korsmeyer-Peppas (R^2^)	0.5823	0.1856
Hixson-Crowell (R^2^)	0.7456	0.4792
Diffusion exponent (n value)	0.2637	0.1856

Supplementary Table S1 indicates variations in the IR peaks between NPf, NPfTX, and NPTX nanoparticles after the loading of paclitaxel. The existence of hydroxyl and amide (–C–OH and NH–), carbonyl (C=O), and ester (C–O–C) groups was observed at the peaks of 3480, 1748, 1100, and 1070 cm^−1^ in the NPfTX spectrum, respectively. These peaks showed the presence of paclitaxel molecules via hydrogen bonding and van der Waals interaction (Fig. 6b,c). The peak observed at 1645 cm^−1^ corresponds to the –C=O– paclitaxel band and indicates the existence of hydrogen bonding (N–H–O–C) between the NPs and paclitaxel (Fig. 6c, see Supplementary Table S1)^49^. Meanwhile, the peaks recorded at 1253 cm^−1^ and 1100–1001 cm^−1^ relate to the paclitaxel –C–O–C– ester band, indicating the drug’s existence in NPfTX (Fig. 6b,iii, see Supplementary Table S1). The peaks at the wavenumber of 922 cm^−1^ showed the –C=C– of the aromatic rings for paclitaxel accordingly^50^. Besides, the –C–H peaks at 808 cm^−1^ in the NPfTX nanoparticles disappeared compared to its existence before drug entrapment, such as in NPf nanoparticles, due to alkyl-alkyl bonded by van der Waals interaction throughout the paclitaxel entrapment process (Fig. 6b,i).

The IR peaks in Fig. 6b(ii) detect the increments and shift at 3402 and 3383 cm^−1^ (O–H and N–H); 1731, 1455, and 1422 cm^−1^ (C=O carbonyl of paclitaxel-nanoparticle interaction); 1646 cm^−1^ (C=O carbonyl of paclitaxel); 1250 and 988 cm^−1^ (C–O–C); and 932 cm^−1^ (aromatic carbon double bond) in order to validate the successful entrapment of paclitaxel in NPTX (see Supplementary Table S1)^51^.

### In vitro drug release, drug release kinetics and mechanism

In vitro drug release study showed that the NPfTX and NPTX nanoparticles had burst release for 4 h from 0 to 75% for NPfTX, and 6 h from 0 to 45% for NPTX. This was followed by a continuous release up to 24 h due to the loss of paclitaxel localised in the nanoparticle matrix from 75% (i.e. 6 h for NPfTX) and 45% (i.e. 8 h for NPTX) to 100% (Fig. 6d). The initial burst release in NPfTX can occur due to the heterogeneous drug distribution (see Fig. 6d), whereas in NPTX, this may present through the pores and cracks associated with the changes in particle morphology particularly related to biodegradability (see Fig. 6d)^52^. In both NPfTX and NPTX, the paclitaxel was fully released in the PBS solutions by the end of 24 h. Therefore, the percentage paclitaxel release practically remained constant between the duration of 24–72 h.

Table 2 shows the yield, drug loading content, the drug entrapment efficiency, the in vitro drug release kinetic values, and the diffusion exponent values of in the NPfTX and NPTX samples, respectively. The drug loading content and drug entrapment efficiency for the NPs were measured using the paclitaxel calibration curve in the DMSO solution (Eq. 6) and calculated using Eqs. (7), (8) and (9). The results revealed that the NPTX nanoparticles had a high nanoparticle yield and less paclitaxel loading capacity at 2.9% compared to the NPfTX nanoparticles. In contrast, the latter had a better loading capacity of 7.12%. Furthermore, NPTX nanoparticles at the hydrodynamic diameter of 176.99 nm were found to be smaller in size compared to NPfTX nanoparticles at the hydrodynamic diameter of 263.08 nm. Therefore, the NPfTX nanoparticles’ peptides may be responsible for the non-covalent interactions between paclitaxel and nanoparticle too due to the existence of functional groups i.e. hydroxyl (–OH), amide (–NH), carbonyl (–C=O) and alkyl (–CH_3_) (see Fig. 5b). This allows additional loading areas for the paclitaxel molecules to the NPfTX’s peptide in comparison to non-peptide-functional nanoparticles, such as in NPTX^49^.

Besides, Table 2 shows that these NPs yield fair linearity for the zero-order, Korsmeyer-Peppas, and Hixson-Crowell kinetics. The best fit with higher correlation was found with the first-order model for the NPfTX nanoparticles, while the Higuchi model applied for the NPTX nanoparticles as the correlation (R^2^) showed high linearity. Based on these findings, the drug release kinetics from the designated NPfTX nanoparticles corresponded to the first-order kinetics. Here, the release frequently starts with an initial burst of a drug, which is shown in the early period of paclitaxel release as seen in Fig. 6d (NPfTX)^53^. For the NPTX nanoparticles (Fig. 6d), the paclitaxel release pattern was best fitted with the Higuchi model based on the higher correlation, indicating the drug release kinetic to be through diffusion control^54^. According to the Korsmeyer–Peppas equation, if the n value is less than 0.45, the drug release follows a quasi-Fickian mechanism. Therefore, the results indicated that the paclitaxel release mechanism from the investigated core–shell NPs (i.e. NPfTX and NPTX) was through diffusion control^55^.

### In vitro cytotoxicity assay

**Figure 7:**
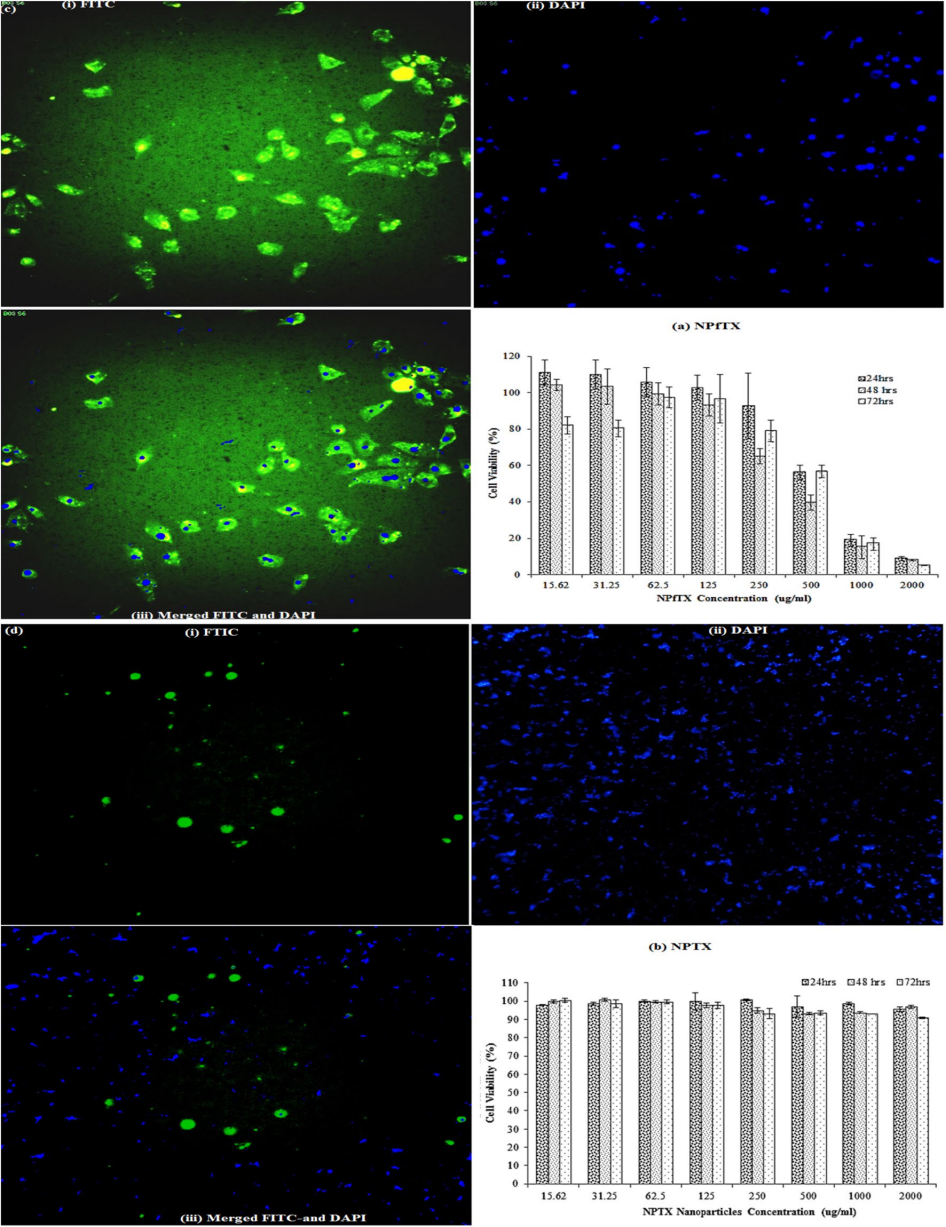
Cell viability of paclitaxel-loaded nanoparticles (**a**) NPfTX and (**b**) NPTX; (**c**) High-content screening of MCF-7 cells with NPfTX nanoparticles after 24 h of incubation. Cellular uptake visualised by overlaying the images collected from stained FITC NPfTX nanoparticles in green (i) stained DAPI cells in blue (ii) and merged FITC channels and DAPI channels (iii); (**d**) High-content screening of MCF-7 cells with NPTX nanoparticles after 24 h of incubation. Cellular uptake visualised by overlaying the images collected from stained FITC NPfTX nanoparticles in green (i) stained DAPI cells in blue (ii) and merged FITC channels and DAPI channels (iii).

Figure 7 indicates the in vitro viability of (a) NPfTX and (b) NPTX, and NPs (Supplementary Fig. S1) samples at the corresponding nanoparticle concentrations of 15.62, 31.25, 62.50, 125, 250, 500, 1000 and 2000 μg/ml. The NPfTX nanoparticles showed both dose- and time-dependent responses (Fig. 7a). Accordingly, the cell viability decreased gradually along with increased nanoparticle dose and incubation time. In particular, NPfTX shows a higher cytotoxicity efficacy against MCF-7 cells than NPTX (see Fig. 7b) and neat NPs (Supplementary Fig. S1). The cell viability of NPfTX-treated MCF-7 cells, on the other hand, was observed to be higher than 100% at low concentrations due to a natural variation in cell metabolism where it is often possible that cell treatment may lead to an increase in enzymatic activity without directly influencing the cell number or cell viability.

Additionally, it can be concluded from Fig. 7a that the MCF-7 cells are inhibited by a sample of peptide-functionalised and paclitaxel-loaded nanoparticles after 24, 48, and 72 h (NPfTX, Fig. 7a). This was due to the presence of peptides that could contribute to the therapeutic action of the NPfTX nanoparticles. Subsequently, the best target for MCF-7 cells and the unique release of paclitaxel molecules was to inhibit the MCF-7 cells, which could be as small as 19.01% and 8.81% cell viability at 1000 and 2000 μg/ml of NPfTX samples after 24 h of incubation, respectively. At 24 h, nearly 100% of the paclitaxel release equal to 53.33 nM of paclitaxel was able to suppress the MCF-7 cells below 19%. Besides, it is suggested that an increased cytotoxicity results in an increased paclitaxel exposure at 48 or 72 h, with 50 nM of paclitaxel in MCF-7 cells^56^. Furthermore, Fig. 7a shows that the MCF-7 cell viability is between 15.21–16.98 and 7.97—5.04% for the NPfTX sample after 48 and 72 h of incubation at 1000 and 2000 μg/ml concentrations, respectively.

No difference in cytotoxicity was observed in NPTX despite the presence of paclitaxel in the assay at 24, 48, and 72 h (Fig. 7b). The NPTX and NPs (Supplementary Fig. S1) samples displayed a moderate cytotoxicity efficacy against MCF-7 cells after 72 h, which reached 90.89% and 80.13% cell viability, respectively. This can be due to the paclitaxel release from NPTX nanoparticles and the degradation components of their matrix, as well as the NP matrix that may inhibit or damage the effects of the MCF-7 cells. This finding is consistent with the biodegradability studies conducted regarding NPs; once they are degraded, these NPs lead to drug leaching and the generation of degradation products that can harm MCF-7^57^. In the paclitaxel release study, NPTX revealed excellent paclitaxel release of about 100%, which was equivalent to 40 nM of paclitaxel concentration for 24 and 48 h. However, even though it was released from NPTX, the MCF-7 cells seemed viable (see Fig. 7b). This finding showed that NPTX acted as a non-target carrier when the absence of peptide in NPTX nanoparticles inhibited NPs targeting MCF-7 cells.

### Cells imaging

Figure 7c,d show the high-content screening images of MCF-7 cells at 24 h of incubation with NPfTX and NPTX nanoparticle dispersion in PBS. The images were taken from: (1) the enhanced green fluorescent protein (FITC) channel (green), (2) the 4′,6-diamidino-2-phenylindole (DAPI) channel (blue), and (3) the two channels merged^58^. From these images, the MCF-cell (blue) can be seen to be located closely around NPfTX nanoparticles (i.e. green, FITC stained), suggesting that they have internalised into the NPs^40^. As a result, the incubation of MCF-7 with NPfTX nanoparticles led to a significant decrease in cell viability, showing there capable of inhibiting cell viability as shown in Fig. 7c^40,58^.

At 24 h, NPfTX demonstrated a 100% release profile equal to 52.5 nM of paclitaxel concentration, suggesting 9–19% inhibition of MCF-7 cells compared to NPTX nanoparticles, where MCF-7 cells were located in NPfTX that had many that were not viable (see Figs. 6d, 7a]. This was due to the high affinity of the peptide-functionalised and paclitaxel binding to NPfTX, thereby causing the high content screening images to reveal the localisation of the MCF-7 cells in the NPfTX^58^.

Figure 7d displays the high-content screening images of MCF-7 cells at 24 h of incubation with NPTX dispersion in PBS. From this figure, within 24 h, only a fewer number of cells (blue) were spread around the NPTX nanoparticles (i.e. green, FITC stained). NPTX also showed a 100% release profile at 24 h, which was equivalent to 40 nm of paclitaxel concentration, but demonstrated almost 100% viable cells compared to NPfTX (see Figs. 6d and 7b). This result revealed that NPTX behaved as the non-target form carriers, whereby these MCF-7 cells could not enter the NPTX nanoparticles. This is shown in the FITC and DAPI merged channels at 24 h in Fig. 7d. The absence of peptide in the NPTX nanoparticles did not significantly affect MCF-7 cells. As a result, the incubation of MCF-7 cells with NPTX nanoparticles did not result in decreased cell viability as shown in Fig. 7b. A slight decrease may be otherwise due to the product degradation and the leaching of the drugs from the NPTX nanoparticles that can affect the MCF-7 cells^57^.

## Conclusions

The production of poly(APO-*b*-polyol ester) nanoparticles by using gamma radiation-induced RAFT polymerisation technique is found to be not only promising and well-suited; the absence of initiators and catalysts in the renders it an environmentally-friendly method for producing the targeted NPs. Besides having a hydrodynamic particle diameter of less than 200 nm when subjected to a very short gamma irradiation exposure (i.e. 700 Gy), the hydrolysed ester bond of these poly(APO-*b*-polyol ester) nanoparticles were found to have very good biodegradable properties: an average of 24 kDa of MW, a controlled MW distribution, and a narrow PDI of 1.01. The study shows that poly(APO-*b*-polyol ester) nanoparticles are competent to be modified with peptide and loaded with paclitaxel in developing active-targeting NPs. The localisation of these MCF-7 cell lines into the cytoplasm of the activated-targeted poly(APO-*b*-polyol ester) nanoparticles revealed the efficacy of these NPs at 1000 and 2000 μg/ml concentrations across specific deliveries. Here, the percentage of cell viability for MCF-7 inhibition could be at 5–20% over a 24, 48 to 72 h of incubation. The cells were found viable without ligands as a targeting agent for NPs due to the non-specific distribution of paclitaxel to cancer cells. As a result, NPTX nanoparticles tended to act as the passive targeting NPs; in comparison, NPfTX acted as the active targeting NPs. Collectively, they exhibited promising properties for binding and destroying cancer cells.

## Experimental

### Synthesis of poly(APO-*b*-polyol ester) nanoparticles

Acrylated palm olein (APO) (molecular weight: 1750.04 g/mol) and polyol ester (molecular weight: 5001.86 g/mol) were synthesised at the Laboratory of Radiation Processing Technology Division, Malaysian Nuclear Agency, Selangor, Malaysia. The macro-APO microemulsion consisting of APO, S,S-dibenzyl trithiocarbonate (DBTTC) (97%, Aldrich), ethyl acetate (EA) (99.5%, Merck), and N,cetyl-N,N,N-trimethylammonium bromide (CTAB) (98%, Merck) were used accordingly without further purification. They were exposed to 500 Gy (Gy) of a gamma radiation source. Then, approximately 20 ml of macro-APO-RAFT solution was added to 3 mg of polyol ester containing 1 mg of DBTTC to obtain the respective poly(APO-*b*-polyol ester) NPs. The mixture was primarily stirred at 300 rpm for 1 h using a magnetic stirrer before being continuously stirred for 1 h using a high-speed disperser at 6000 rpm. The mixture was degassed with nitrogen gas before being exposed to a range of gamma radiation doses (i.e. 100, 400, and 700 Gy, and 1, 5, and 10 kGy). All samples were irradiated by using gamma radiation from a Cobalt-60 source at a dose rate of 2.16 Gy/s.

### Physicochemical properties

The hydrodynamic mean diameter (nm) of the samples was determined by photon cross-correlation spectroscopy (PCCS) using DLS (Sympatec Nanophox, German) with a helium–neon (HeNe) laser at a wavelength of 632 nm. The sample charge was analysed at room temperature via a zeta potential analyser (Zetapals, Brookhaven, USA). The palladium electrode cell was fitted and immersed into a 1 cm quartz cell consisting of the test solution. The zeta potential of the sample was determined using phase analysis light scattering (PALS) with a HeNe laser at a wavelength of 632 nm. Images of the NPs were captured by using a transmission electron microscope (TEM) for their morphological properties. Accordingly, the samples were dispersed in ultrapure water and a few droplets were placed on a copper grid to be dehydrated at room temperature. The TEM images were analysed by using a Zeiss microscope (Jeol, Japan) at a voltage of 160 kV. The chemical functional groups of the NPs were analysed by using Spectrum 400, Fourier Transform Near-Infrared (FT-NIR) spectrometer (Perkin Elmer, UK) within the wavenumber range of 500 to 4000 cm^−1^. Next, the samples were dissolved in deuterated chloroform (CDCl3) for the NMR measurement. The _δ_H-NMR spectroscopy was performed by using a Bruker Avance Fourier Transform Nuclear Magnetic Resonance (FT-NMR) Spectrometer (Germany) with a field strength of 600 MHz at room temperature, 28.05 °C and using a line broadening (LB) of 0.30 Hz. Then, GPC (Waters 2414, USA) was utilised to analyse the average molecular weight of samples. Approximately 5 mg of sample was weighed and solubilised in 5 mL of tetrahydrofuran (THF) (99.9%, Merck). The solution was kept for 24 h at room temperature before its measurement. The GPC operation system was set up at 35 °C with 1 mL/min of flow rate using the THF eluent.

### In vitro degradation study

The in vitro degradation of the NPs was performed in an incubator at 37 °C with orbital shaking at 150 rpm for the set periods of 1, 7, 30, 60, and 90 days accordingly. For each degradation period, a sample containing about 1 mg of NPs was weighed and placed in screw cap glass test tubes. Then, 5 ml of the simulated body fluid (SBF) solution (pH 7.4) was added into the test tubes, which was replaced every week to maintain the freshness of the fluid. After a specific period of incubation, the SBF solution was removed from the test tubes. Next, the NPs kept in the test tubes were rinsed with ultrapure water before being freeze-dried and weighed. The degree of biodegradation is calculated based on the weight loss percentage of the NPs using Eq. (1) below:

1 $${\text{Nanoparticles}}\,\,{\text{weight}}\,\,{\text{loss}}\,\,(\% ) = \frac{{{\text{Weight}}\,\,{\text{of}}\,{\text{initial}}\,\,{\text{sample}} - {\text{Weight}}\,\,{\text{of}}\,\,{\text{final}}\,\,{\text{sample}}}}{{{\text{Weight}}\,\,{\text{of}}\,{\text{initial}}\,\,{\text{sample}}}} \times 100$$

The biodegradation of the NPs was determined by comparing the FTIR peak profile of the degraded nanoparticle sample with the fresh sample by an FTIR/FT Near-IR (FT-IR / FT-NIR) spectrometer (Perkin Elmer, UK) within the wavenumber range of 500–4000 cm^−1^. Furthermore, images of the same set of NPs were captured by using a TEM (Jeol, Japan) for their morphological properties at a voltage of 160 kV. The dried nanoparticle samples were dispersed in 1 ml of acetone. A few droplets of the samples were placed on a copper grid and dehydrated at room temperature.

### Surface functionalisation of nanoparticles with peptide

The carboxylic (-COOH) group was functionalised to the poly(APO-*b*-polyol ester) NPs using the succinylation technique. First, 30 mg of NPs was weighed in a 50 ml flask and dissolved with 5 ml of 1,4-dioxane. The sample was stirred for 30 min at 300 rpm. Afterwards, preparation of 0.93 mg of succinyl anhydride, 7.32 mg of 4-Dimethylaminopyridine (DMAP), and 9.18 ml of triethylamine was dissolved in 5 ml of 1,4-dioxane and stirred for 30 min at 300 rpm before being added to a solution of NPs. The mixture was then stirred at 250 rpm and heated at 90 °C for 12 h under nitrogen. The nanoparticle derivatives were collected using the gravimetric method, while the sample was cooled before re-dissolved with 10 ml dimethylformamide (DMF) and precipitated using 10 ml of diethyl ether as precipitant. The sample was centrifuged for 1 h at 5000 rpm and dried under vacuum at room temperature for 24 h to produce the carboxylate NPs.

N-hydroxysuccinimide (NHS) activation with the carboxylic-functionalised NPs was performed by using the NHS and EDC. Accordingly, 15 mg of carboxylic-functionalised NPs and 1.77 mg of NHS were weighed in a 50 ml flask and dissolved with 10 ml of dichloromethane. The solution was stirred for 30 min at 250 rpm. Next, 4.05 mg of EDC was added to the solution. The mixture was mixed at room temperature for 12 h under nitrogen with a stirring speed of 250 rpm. The NPs derivatives were collected using the gravimetric method. The sample was re-dissolved in 5 ml ethyl acetate and precipitated using 10 ml of diethyl ether as a precipitant. The sample was centrifuged for 1 h at 5000 rpm and dried under vacuum at room temperature for 24 h to produce the NHS-activated NPs.

About 100 μL of 0.1 mM of peptide was dissolved in 5 ml of DMF in a 20 ml flask. Next, 1.12 mg of DMAP and 2.72 μL were mixed into the peptide solution. Afterwards, 10 mg of NHS-activated-nanoparticles was dissolved in 10 ml DMF in a 50 ml flask. The mixture of peptide solution was then added to the NHS-activated-nanoparticles solution while stirring at a speed of 250 rpm for 12 h at room temperature under nitrogen. The sample was re-dissolved in 5 ml ethyl acetate and precipitated using 10 ml of diethyl ether as the precipitant, following which it was centrifuged for 1 h at 5000 rpm and dried under vacuum at room temperature for 24 h to produce the peptide-functionalised nanoparticles.

The concentration of the peptide conjugated into the nanoparticles was determined using UV–Vis spectrophotometer (Shidmazu UV-1800, Japan) at wavelengths between 190 and 500 nm and the wavelength at 275 nm was chosen for obtaining the absorbance peak values. The concentration and the percentage of the conjugated peptide were calculated according to the peptide calibration curves using Eqs. (2) and (3), respectively. Meanwhile, the NHS usage was determined via FTIR at wavenumbers between 4000 to 500 cm^−1^ and the wavenumber at 1789 cm^−1^ was chosen for obtaining the absorbance peak ratio values. The concentration and the percentage of NHS usage were calculated according to the NHS calibration curves using Eqs. (4) and (5), respectively.

2 $$y = 0.5592x$$

where x represents the concentration of the peptide in ultrapure water.

3 $${\text{Percentge}}\,\,{\text{of}}\,\,{\text{peptide}}\,\,{\text{conjugated}}\,\,(\% ) = \frac{{{\text{Actual}}\,\,\,{\text{concentration}}\,\,{\text{of}}\,\,{\text{peptide}}\,\,{\text{conjugated }}\,\,({\text{mM}}) - {\text{Excess}}\,\,{\text{concentration}}\,\,{\text{of}}\,{\text{peptide}}\,\,{\text{unconjugated}}\,\,({\text{mM}})\,}}{{{\text{Initial}}\,\,{\text{weight}}\,\,{\text{of}}\,\,{\text{peptide}}\,\,({\text{mM}})}} \times 100$$

4 $$y = 3.2418x$$

where x represents the concentration of the NHS in DCM.

5 $${\text{Percentage}}\,\,{\text{of}}\,\,{\text{NHS}}\,\,{\text{usage}}\,\,(\% ) = \frac{{{\text{Actual}}\,\,{\text{weight}}\,\,{\text{of}}\,\,{\text{NHS}}\,\,{\text{usage (mg/ml)}} - {\text{Excess}}\,\,{\text{weight}}\,\,{\text{of}}\,\,{\text{NHS (mg/ml)}}}}{{{\text{Initial}}\,\,{\text{weight}}\,\,{\text{of}}\,\,{\text{NHS (mg/ml)}}}} \times 100$$

### Paclitaxel encapsulation of nanoparticles

About 2.5 mg of peptide-functionalised-nanoparticles was added in 5 ml of 50 nM of paclitaxel in the DMSO solution. The mixture was stirred for 8 h at 500 rpm. After the entrapment process, the sample was precipitated using 2 ml of diethyl ether as the precipitant and centrifuged for 1 h at 5000 rpm, whereby the resulting supernatant was separated and collected. The sample was purified by dialysis against deionised water with dialysis tubing for 24 h to remove any excess impurities and DMSO. Then, the remaining solution in the dialysis tubing was transferred to a new vial and dried under vacuum at room temperature to produce solid NPs.

The supernatant solution were analysed by using UV–Vis spectrophotometer at the wavelengths between 190 and 500 nm; the maximum UV absorption wavelength at 265 nm was thus chosen to obtain the absorbance peak values^59^. The paclitaxel calibration graph is obtained by plotting the absorbance against the paclitaxel concentrations, while the excess concentration of paclitaxel in the supernatant that is not entrapped is calculated using the paclitaxel calibration curve according to Eq. (6). Afterwards, the actual amount of drug entrapped and loading can be obtained.

6 $$y = 0.0006x + 0.0381$$

where x represents the concentration of the paclitaxel in DMSO.

Subsequently, the yield of NPs produced, drug loading content, and drug entrapment efficiency are calculated using Eqs. (7), (8) and (9). On the other hand, the above procedure was repeated for the production of drug-loaded nanoparticles using non-functional (or neat) nanoparticles.

7 $${\text{Nanoparticles}}\,\,{\text{yield}}\,\,(\% ) = \frac{{{\text{weight}}\,\,{\text{of}}\,\,{\text{nanoparticles}}}}{{{\text{weight}}\,\,{\text{of}}\,\,{\text{polymer}}\,\,{\text{and}}\,\,{\text{drug}}\,\,{\text{fed}}\,\,{\text{initially}}}} \times 100$$

8 $${\text{Drug}}\,\,{\text{loading}}\,\,{\text{content}}\,\,(\% ) = \frac{{{\text{weight}}\,\,{\text{of}}\,\,{\text{drug}}\,\,{\text{in}}\,\,{\text{nanaparticles}}}}{{{\text{weight}}\,{\text{of}}\,\,{\text{nanoparticles}}}} \times 100$$

9 $${\text{Entrapment}}\,\,{\text{efficiency}}\,\,(\% ) = \frac{{{\text{weight}}\,\,{\text{of}}\,\,{\text{drug}}\,\,{\text{in}}\,\,{\text{nanaparticles}}}}{{{\text{weight}}\,\,{\text{of}}\,\,{\text{drug}}\,\,{\text{fed}}\,\,{\text{initially}}}} \times 100$$

### In vitro drug release

Two type NPs namely peptide-functionalised and paclitaxel-loaded poly(APO-*b*-polyol ester) nanoparticles (NPfTX) and paclitaxel-loaded poly(APO-*b*-polyol ester) nanoparticles (NPTX) were used for determining the in-vitro drug release from the nanoparticulate drug delivery systems by using a dialysis membrane method.

First, 2.5 mg of NPs (i.e. NPfTX or NPTX) in 5 ml PBS was transferred to a dialysis tubing and closed tightly. The dialysis tubing was placed in a 50 ml beaker with a magnetic stir bar, whereby 45 ml of PBS was added. Then, the beaker was positioned in the flat bottom beaker containing distilled water that was placed on a hotplate magnetic stirrer. The paclitaxel release was conducted at the set periods of 15, 30, and 45 min and 1, 2, 3, 4, 5, 6, 8, 24, 48 and 72 h accordingly, at a temperature of 37 °C and speed of 250 rpm. A 2 mL sample solution was taken at each time interval and replaced with 2 ml of fresh PBS solution. The solution was then analysed by using UV–Vis spectrophotometer at wavelengths between 190 and 500 nm, whereby the wavelength at 265 nm was chosen to obtain the absorbance peak values. Next, the concentration of paclitaxel release from the NPs was calculated using the paclitaxel calibration curve in PBS solution as shown in Eq. (10), while the cumulative percentage of paclitaxel released was calculated using Eq. (11). The concentration study of paclitaxel release from NPs was performed in a triplicate with all data expressed as the mean (± standard deviation). Additionally, the kinetics and drug release mechanism were determined by the use of in vitro release data for different models of kinetics.

10 $$y = 0.0004x$$

where y is absorbance value; x is concentration of paclitaxel release.

Cumulative percentage of paclitaxel release

11 $$y = \frac{{w_{t} }}{{w_{c} }} \times 100$$

where W_c_ is the total paclitaxel concentration in the dialysis membrane and W_t_ is the paclitaxel concentration in the PBS medium at time, ‘t’.

### In-vitro cytotoxicity

The initial stock solutions of the NPfTx and the NPTx samples were made at 2000 μg/ml of PBS in a 15 ml of centrifuge tube, and then vortexed at room temperature. The serial dilutions of these nanoparticle solutions were prepared at a final volume of 2 ml in the 12-well culture plate by using a complete media solution within the concentration range from 15.63, 31.25, 62.5, 125, 250, 500, 1000 to a 2000 μg/ml. These nanoparticle serial dilution solutions were used in the 3-(4,5-dimethylthiazol-2-yl)-2–5-diphenyltetrazolium bromide (MTT) assay.

First, 100 μl of complete media solutions was aspirated from the incubated MCF-7 cells with a concentration of 7.5 × 10^3^ cells in a 96-well culture plate. Then, each of the 100 μl nanoparticle serial dilution solutions were seeded to the incubated MCF-7 cells, respectively. The 96-well culture plates were next incubated at 24, 48, and 72 h in a 5% carbon dioxide (CO_2_) incubator system.

Next, 20 μl of 5 mg/ml of MTT reagent was added to the MCF-7 cells accordingly. These 96-well culture plates were kept for 4 h in a 5% CO_2_ incubator system. Afterwards, all of the complete media solutions were removed from the MCF-7 cells and 100 μl of DMSO solution was pipetted to the 96-well culture plates, respectively. Then, the culture plates were shaken for 10 min at room temperature in a dark place and placed in the multimode plate reader (Enspire) to perform the MTT assay. The absorbance of the cells was measured at a wavelength of 540 nm by using the Enspire manager software. Then, the cellular viability for the set nanoparticle serial dilution concentrations (i.e. 15.63, 31.25, 62.5, 125, 250, 500, 1000 to a 2000 μg/ml) was calculated following Eq. 12 and the values were plotted in graphs accordingly. The MCF-7 cell viability analysis regarding the effect of paclitaxel release from the NPs was conducted in a triplicate of all data expressed as the mean (± standard deviation).

Calculation on the cell viability:

12 $${\text{Cell}}\,\,{\text{viability}}\,,\,\% = \frac{{{\text{Absorbance}}\,\,{\text{of}}\,\,{\text{sample}}}}{{{\text{Absorbance}}\,\,{\text{of}}\,\,{\text{blank}}}} \times 100$$

### Cells imaging

An N-Hydroxysuccinimide (NHS)-fluorescein was used for staining the NPfTX and NPTX NPs at a half-maximum inhibitory concentration (IC50) value of 500 μg/ml followed the IC50 value of the NPfTx NPs interacting with MCF-cells at 24 h. The test was conducted following the Thermo Scientific standard protocol method (2082.1 46409 46410) for NHS-Fluroscein antibody labeling^60^. A total of 2000 μl solution composed of 17.24 μl of 2.55 × 10^–5^ mmol of NHS-fluorescein, a 210 μl of NPfTX solution, a 160 μl of conjugate buffer, and a 1613 μl of complete media was prepared in a 2 ml microcentrifuge tube for staining the NPfTX NPs. For staining the NPTX NPs, a total of 2000 μl solution composed of 5.10 μl of 7.53 × 10^–6^ mmol of NHS-fluorescein, 52.80 μl of nanoparticle solution, 160 μl of conjugate buffer, and 1782 μl of complete media was prepared in a 2 ml microcentrifuge tube.

These stained nanoparticle solutions were vortexed and incubated for 1 h at room temperature. Afterwards, any excess NHS-fluorescent dye in the stained NPs was removed using the dialysis technique and the figures of the experimental setup were displays in Supplementary Fig. S2. The sample solution was transferred to a single Slide-A-Lyser MINI Dialysis unit, which was floating in a dark container with at least 100 ml of PBS to protect the NHS from light. The samples were dialysed for 24 h at room temperature. Subsequently, the sample solutions were removed from the dialysis unit and transferred to a 2 ml microcentrifuge tube. These sample solutions composed of NHS- fluorescein stained NPs were used for cellular imaging by using the Image Xpress Micro High Content Screening (Molecular Devices) system.

## Supplementary Information

Supplementary information

## Data Availability

All data generated or analysed during this study are included in this published article (and its Supplementary Information files).
