# Supplementary material for: Surface functionalisation of poly-APO-b-polyol ester cross-linked copolymers as core–shell nanoparticles for targeted breast cancer therapy
Source: Sci Rep. 2020 Dec 10;10:21704. doi: 10.1038/s41598-020-78601-x (PMC7729971; doi:10.1038/s41598-020-78601-x)
Supplement: Supplementary file 1 — Supplementary information [file 41598_2020_78601_MOESM1_ESM.pdf]

Supplementary information

Surface functionalisation of poly-APO-*b*-polyol ester cross-linked copolymer as a core-shell of nanoparticles for targeted breast cancer therapy

Rida Tajau, [1,2] *Rosiah Rohani *[1], Siti Selina Abdul Hamid[3], Zainah Adam[3], Siti Najila Mohd Janib[3] and Mek Zah Salleh[2]*

[1]*Department of Chemical & Process Engineering, Faculty of Engineering and Built Environment, Universiti Kebangsaan Malaysia, 43600 UKM Bangi, Selangor, Malaysia.*
[2]*Division of Radiation Processing Technology, Malaysia Nuclear Agency, Bangi, 43000 Kajang, Selangor, Malaysia.*
[3]*Division of Medical Technology, Malaysia Nuclear Agency, Bangi, 43000 Kajang, Selangor, Malaysia.*
**Corresponding author: rosiah@ukm.edu.my*

Table S1 Assignment of FTIR peak between peptide-functionalised nanoparticles in comparison with NHS-activated nanoparticles.

Functional group	Assignment IR peak (cm^{-1})				
	Macro-APO-RAFT agent	Poly(APO-*b*-polyol ester) nanoparticles	Carboxylated nanoparticles	NHS activated nanoparticles	Peptide-functionalised nanoparticles
-OH	3441	3459	3494	3379	3491
-CH	2923,2853	2923,2854	2968,2933,2860	2941, 2878	2941, 2884, 2848
Thiol (SH)	2658	2689	2807	2798	2798
N=C=S stretching isothiocyanate	2139,2084	2140, 2020	2141	2123	2123
C=O	1741	1742	1739	1789	-
C=O of COOH	-	-	1709	1715	-
N-O (nitro)	-	-	-	1645	-
-OH	-	-	1623	-	
Aromatic C=C bending	1675,1632,1603	1588,1562	1591	-	-
N-H	-	-	-	1,585	1645
C-H	-	-	1473,1422	1473, 1395	1472,1390
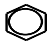	1395,1399	1380	1379	-	1370
C-O	1170,1134,1077, 1058	1171,1120			
C-O-C	-	-	1246, 1170, 1113, 1097, 1058	1233, 1197, 1179, 1106, 1040, 988	1239, 1136, 1103
C-O-O	1495,1455	1465,1448	-	-	-
C=S	1241	1250	-	-	-
C-N	-	1000, 1028	1057	1020	1040
Aromatic C=C bending	988,950,855	940	-	-	-
N-H	-	-	-	879	872
C-H	795,786,726,698	726	801,734,699	801,741,699	798,708,709

Table S2 ^{1}H-NMR of macro-APO-RAFT agent, poly(APO-*b*-polyol ester) nanoparticles and Npf nanoparticles.

Type of Proton	Approximate Chemical Shift (ppm)					
	ID No.	Macro-APO-RAFT	ID No.	Poly(APO-*b*-polyol ester) nanoparticles	ID No.	NPf
$-CH_3$	1	0.798-0.821	1	0.874-0.897	1	0.900
$-CH_2$	2	1.183-1.214	2	1.260-1.290	2	1.259
-CH	3	1.492-1.535	3	1.400-1.500	3	1.490-1.502
$-CH_2$-S-(C=S)-S- (Thio Sulfonate)	4	1.672	4	1.612-1.796, 1.682-1.849	4	1.641, 1.722-1.746,2.023-2.044
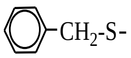 (sulfide)	5	1.895-1.944, 3.426-3.529	5	1.895-1.944	5	2.091-2.117
$-CH_2$-N		-	6	2.100-2.200	6	2.182
$-CH_2$C(O)O-	6	2.025, 2.229-2.309	7	2.305-2.329	7	2.300-2.400
-C=O-OH		-		-	8	2.744
$-NH_2$		-		-	9	2.854
-NH		-		-	10	2.998
$-CH_2$-OH		-		-	11	3.257
-OH	7	2.60-2.80	8	2.60-2.80		-
-CH-OH	8	3.652	9	3.500-3.600		-
-COO-CH_2-		-	10	3.700-3.950		-
$-CH_2$CH-C(O)O-	9	4.062-4.230	11	4.139-4.307	12	4.358-4.393
-CH-O-C=O-	10	4.552	12	4.900-4.800	13	4.600-4.700
-CH-O-C(O)-	11	5.188-5.309	13	5.100-5.200		-
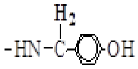		-		-	14	5.136-5.171
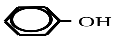		-		-	15	6.325, 6.716-6.728
-C=O-NH-		-		-	16	7.10
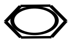	12	7.270-7.265	14	7.300-7.500	17	7.300-7.400
-NH-C=O-		-		-	18	8.159-8.181

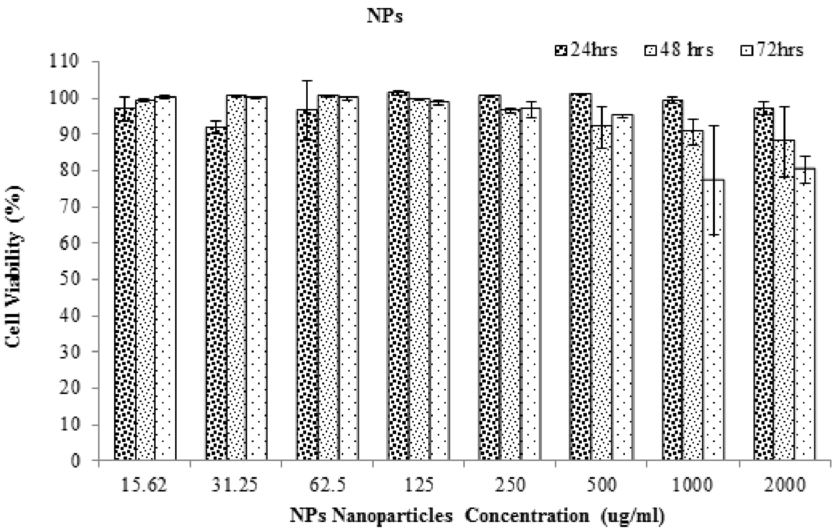

Figure S1 Cell viability of neat nanoparticles (NPs).

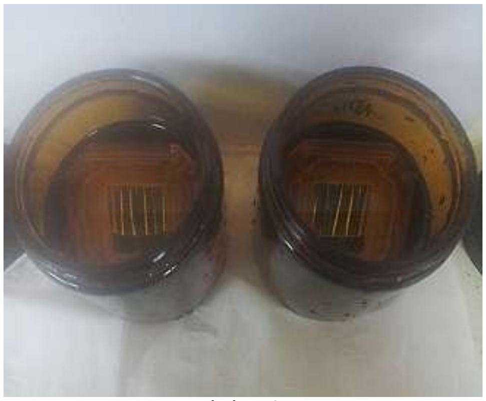

(a) 0 h

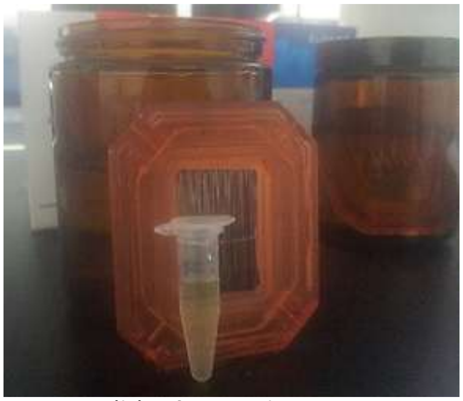

(b) After 24 h

Figure S2 NHS-fluorescein stained nanoparticles dialysis with dialysis cassettes in PBS solution.
